# Hypoxia-Related Gene FUT11 Promotes Pancreatic Cancer Progression by Maintaining the Stability of PDK1

**DOI:** 10.3389/fonc.2021.675991

**Published:** 2021-06-17

**Authors:** Wenpeng Cao, Zhirui Zeng, Runsang Pan, Hao Wu, Xiangyan Zhang, Hui Chen, Yingjie Nie, Zijiang Yu, Shan Lei

**Affiliations:** ^1^ Department of Anatomy, School of Basic Medicine, Guizhou Medical University, Guiyang, China; ^2^ Department of Physiology, School of Basic Medicine, Guizhou Medical University, Guiyang, China; ^3^ Department of Orthopedics, Guiyang Maternal and Child Health Care Hospital, Guiyang, China; ^4^ Department of Pediatric Surgery, The Affiliated Hospital of Guizhou Medical University, Guiyang, China; ^5^ NHC Key Laboratory of Pulmonary, Guizhou Provincial People’s Hospital, Guiyang, China; ^6^ The Clinical Lab Center, Guizhou Provincial People’s Hospital, Guiyang, China

**Keywords:** pancreatic cancer, hypoxia, fucosyltransferase 11, hypoxia-inducible factor 1α, pyruvate dehydrogenase kinase 1

## Abstract

**Background:**

Hypoxia is associated with the development of pancreatic cancer (PC). However, genes associated with hypoxia response and their regulatory mechanism in PC cells were unclear. The current study aims to investigate the role of the hypoxia associated gene fucosyltransferase 11 (FUT11) in the progression of PC.

**Methods:**

In the preliminary study, bioinformatics analysis predicted FUT11 as a key hypoxia associated gene in PC. The expression of FUT11 in PC was evaluated using quantitative real-time PCR (qRT-PCR), Western blot and immunohistochemistry. The effects of FUT11 on PC cells proliferation and migration under normoxia and hypoxia were evaluated using Cell Counting Kit 8, 5-ethynyl-2’-deoxyuridine (EDU) assay, colony formation assay and transwell assay. The effects of FUT11 *in vivo* was examined in mouse tumor models of liver metastasis and subcutaneous xenograft. Furthermore, Western blot, luciferase assay and immunoprecipitation were performed to explore the regulatory relationship among FUT11, hypoxia-inducible factor 1α (HIF1α) and pyruvate dehydrogenase kinase 1 (PDK1) in PC.

**Results:**

FUT11 was markedly increased of PC cells with hypoxia, upregulated in the PC clinical tissues, and predicted a poor outcome of PC patients. Inhibition of FUT11 reduced PC cell growth and migratory ability of PC cells under normoxia and hypoxia conditions *in vitro*, and growth and tumor cell metastasis *in vivo*. FUT11 bound to PDK1 and regulated the expression PDK1 under normoxia and hypoxia. FUT11 interacted with PDK1 and decreased the ubiquitination of PDK1, lead to the activation of AKT/mTOR signaling pathway. FUT11 knockdown significantly increased the degradation of PDK1 under hypoxia, while treatment with MG132 can relieve the degradation of PDK1 induced by FUT11 knockdown. Overexpression of PDK1 in PC cells under hypoxia conditions reversed the suppressive impacts of FUT11 knockdown on PC cell growth and migration. In addition, HIF1α bound to the promoter of FUT11 and increased its expression, as well as co-expressed with FUT11 in PC tissues. Furthermore, overexpression of FUT11 partially rescued the suppressive effects of HIF1α knockdown on PC cell growth and migration in hypoxia condition.

**Conclusion:**

Our data implicate that hypoxia-induced FUT11 contributes to proliferation and metastasis of PC by maintaining the stability of PDK1, thus mediating activation of AKT/mTOR signaling pathway, and suggest that FUT11 could be a novel and effective target for the treatment of pancreatic cancer.

## Background

Pancreatic cancer (PC) has high morbidity and mortality worldwide (1). Although the treatment for PC such as surgery, targeted therapy and chemotherapy had been improved, the number of PC related mortality is still increasing every year (2). Therefore, it is a pressing need to uncover the molecular mechanism involved in PC and explore the potential biomarkers for diagnosis and as novel targets for treatment of PC.

The tumor microenvironment plays a vital role in the development of tumors and is closely related to the efficacy of tumor treatment. Targeting the tumor environment is a therapeutic strategy for cancer treatment (3). Generally, hypoxic microenvironment of tumors up-regulates a series of hypoxic-responsive genes, and induces the proliferation, migration, drug resistance and other biological events of cancer cells (4). Hypoxia inducible factor 1-alpha (HIF1α) is a main regulator of transcriptional response to hypoxia in cancer cells. HIF1α up-regulates a number of genes that support tumor cells to adopt to the hypoxic microenvironment (5). HIF1α overexpression has been detected in solid tumors and is associated with the progression of a variety of cancers, including ovarian cancer (6), breast cancer (7), non-small cell lung cancer (8) and pancreatic cancer (9). Studies have shown that HIF1α affects the regulation of tumor cell proliferation, angiogenesis, apoptosis and chemotherapy resistance during tumor development (10). However, the target genes of HIF1α in PC remain to be elucidated.

Fucosyltransferases (FUTs) are a family of enzymes which catalyzed the transfer process of fucose from GDP-fucose to glycoconjugates (11). Previous studies have demonstrated that the FUT family is closely related to the occurrence and development of tumors. Liang et al. demonstrated that miR-125a-3p/FUT5-FUT6 axis mediates the proliferation, mobility and pathological angiogenesis of colorectal cancer through the PI3K-Akt pathway (12). Kumar et al. showed that NCOA3 stabilized mucins post translationally through FUT8, which promoted the proliferation and metastasis of pancreatic cancer (13). Lin et al. reported that FUT11 and FUT1 genes were down-regulated in cisplatin-resistant cells (14). However, the functions and regulatory mechanisms of FUT11 in PC remain largely unclear.

In this study, we found that the FUT11 was a direct target gene of HIF1α by bioinformatics analysis and it was up-regulated in PC cells under hypoxia. FUT11 promoted the proliferation and metastasis of PC cells *via* maintaining the stability of pyruvate dehydrogenase kinase 1(PDK1) under hypoxia. Our study indicates FUT11 could be a therapeutic target for the treatment of PC.

## Methods

### Bioinformatics Analysis

We downloaded the Gene expression profile GSE67549 and GSE9350 from the Gene Expression Omnibus database (GEO, https://www.ncbi.nlm.nih.gov/gds). GSE67549 contained 9 normoxic PC cell samples and 9 hypoxic PC cell samples, while GSE9350 contained 2 normoxic PC cell samples and 2 hypoxic PC cell samples. Differential expression genes were identified using the cut-off as Log2 fold change (FC) >1 and P value <0.05. Common differential expression genes were analyzed using intersection analysis. The expression of these genes in the PC samples of The Cancer Genome Atlas (TCGA) and The Genotype-Tissue Expression (GTEx) database was determined using GEPIA (http://gepia.cancer-pku.cn/). *P*<0.05 was a threshold to be considered as statistically significant.

### Clinical Samples

A total of 90 paired PC tissues and adjacent pancreatic tissues were obtained from PC patients who had surgery in the Affiliated Hospital of Guizhou Medical University. None of them received radiotherapy or chemotherapy prior to the surgery. The current study was approved by the Ethics Committee of Guizhou Medical University in accordance with the Declaration of Helsinki, and all patients who participated in the current study signed their informed consents.

### Cell Culture and Transfection

Two human PC cell lines (PANC-1 and AsPC-1) used in the current study were obtained from ATCC. PANC-1 and AsPC-1 cells were cultured in DMEM with 10% FBS at 37˚C with 5% CO2. The condition of normoxia was set to 21% O_2,_ 74% N_2_ and 5% CO_2_, while the condition of hypoxia was set to 1%O_2_, 94%N_2_ and 5%CO_2_. Oligonucleotides targeting FUT11 (GUUAGAGACCACUGUAUCUGC) were cloned into the pLKO.1 vector (GenePharma, Shanghai, China). Full‐length PDK1 coding sequence was subcloned into the lentiviral vector pCD315B-1 (System Biosciences, Beijing, China). The small interfering RNA (siRNA) targeting HIF1α were obtained from JIMA (Shanghai, China). To construct the stable cell lines with target gene overexpression or knockdown, 1 μg/mL puromycin (Sigma, USA) was added to culture medium after transfection with lentivirus for 48h to continuously screen the stable cells for 10 days.

### Quantitative Real-Time PCR (qRT-PCR)

Total RNA in PC tissues and cells was separated using TRIZOL reagents (Beyotime Biotechnology, Hangzhou, China) and diluted into DNase/RNase-free water. After quantification, total RNA (2µg per sample) was reversely transcribed into cDNA using RevertAid First Strand cDNA Synthesis Kit (Fermentas, USA). Finally, quantitative real-time PCR (qRT-PCR) was conducted to determine the expression level of target genes using SYBR™ Green PCR Master Mix (Solarbio, Wuhan, China). β-actin was used as the internal control. The primer sequences in our study were purchased from Tianyi Huiyuan (Wuhan, China) and shown in **Supplementary Table 1**.

### Cell Counting Kit-8 (CCK-8) Assay

PANC-1 and AsPC-1 cells were plated in a 96-well plate in sextuplicate with 3×10^3^ cells/well. Briefly, 100μl DMEM medium containing 10μl CCK-8 regent (Boster, Wuhan, China) was added to each well in 24h, 48h, 72h and 96h. The light absorbance of each well was detected at 450nm.

### 5-ethynyl-2’-deoxyuridine (EDU) Assay

The EDU assay was carried out using a BeyoClick™ EdU-488 Proliferation Detection Kit (Beyotime, Suzhou, China). In brief, PC cells were cultured in 6-well plates and were allowed to adhere. The primary culture medium was removed and fresh medium was added. Then, 10μM EDU was added into each well and cells were cultured in 37°C for 2.5h. After that, cells were fixed in 4% paraformaldehyde (Beyotime, Suzhou, China) for 15 min and permeabilization using 0.3% Triton X-100 (Boster, Wuhan, China) for 8 min. Then, 500μl Apollo dyeing reaction buffer was added for 40 min in the dark. After staining, the nuclei were stained using DAPI for 10 min. The EDU staining was observed under a fluorescence microscope (Zeiss, Oberkochen, Germany).

### Colony Formation Assay

Cells with a density of 2000 cells/well were seeded into 6-well plates and cultured in DMEM media containing 10% FBS. After 24h, intervention factor was added and cultured for 2 weeks. After fixation in 4% paraformaldehyde for 15 min, 1% crystal violet was used to stain cell colonies. Cell colonies was counted and photographed.

### Western Blotting

The proteins in PC cells and tissues were extracted using RIPA reagent contain 5% PMSF protease inhibitor. The BCA method was performed to examine the protein concentration of each sample. Proteins (30μg/per line) were added and separated by 10% SDS-PAGE for 120 min. Then, the proteins were transferred into the PVDF membranes (Millipore, USA) with 0.45μm pore diameter, which was then blocked in 5% BSA for 30min and incubated with primary antibodies including FUT11 (Abcam, cat. no. ab121411, dilution, 1:500), N-cadherin (CST, cat. no. 14215, dilution, 1:1000), E-cadherin (CST, cat. no. 3195, dilution, 1:1000), PDK1 (Santa, cat. no. 4A11F5, dilution, 1:1000), AKT (CST, cat. no. 9272, dilution, 1:1000), p-AKT (CST, cat. no. 9271, dilution, 1:1000), mTOR (CST, cat. no. 2972, dilution, 1:1000), p- mTOR (CST, cat. no. 2971, dilution, 1:1000), HIF1α (CST, cat. no. 36169, dilution, 1:1000) and β-actin (CST, cat. no. 3700, dilution, 1:1000) for 12h in 4˚C. High sensitivity ECL reagent was used to visualize the blots in MultiImager and the relative expression of protein was calculated using Image J. β-actin was set as reference for FUT11, N-cadherin, E-cadherin, PDK1 and HIF1α.

### Transwell Assay

For transwell migration assay, a total of 1×10^5^ cells were suspended using 200μl DMEM medium without FBS and seeded into the upper transwell chambers (Becton, Dickinson and Company, USA). Total 600μl DMEM medium contained 10% FBS was placed in the lower transwell chambers. After 24h, migratory cells were fixed with paraformaldehyde and stained using 0.5% crystal violet. Finally, the migratory cells were counted and photos were taken.

### *In Vivo* Assay

For subcutaneous tumor xenograft model, 10 female BALB/c nude mice were obtained from the animal central of Guizhou Medical University (Guizhou, China). After adaptive feeding, a total of 1×10^6^ PANC-1 cells with FUT11 knockdown and negative control cells were subcutaneously injected into the upper-right flank of BALB/c mice (n = 5 in each group). The health status of mice was monitored every day. The tumor volume was monitored once a week and determined as followed: (mm^3^) = (Long×Width^2^)/2. After 5 weeks, the mice were sacrificed and tumor tissues collected. The protein level of KI67 and PCNA in tumor tissues was determined using immunohistochemical staining. The liver metastatic tumor model was established by injecting the FUT11 knockdown and negative control PANC-1 cells into the spleen capsule. FUT11 knockdown and negative control group PANC-1 cells (1×10^7^ cells) were injected into the spleen of BALB/c mice (n=5 in each group). Animal health and behavior after injecting were monitored each day. While the mouse had the features of hard breath and limitation of motion, mice were sacrificed in order to reduce animal suffering, and the liver tissues were dissected and used to count the metastatic foci. While mice in one group were all sacrificed, the animal experiment was terminated and the rest of mice were all euthanasia. Finally, unpair-t test was used to determine the significant between this group according to the number of metastasis foci. HE staining was also used to detect the condition of metastatic foci in the liver. All procedures of animal studies were approved by the Ethics Committee of Guizhou Medical University and followed the legal mandates and national guidelines for the care and maintenance of laboratory animals.

### Immunofluorescence Staining

Cells were fixed with 4% paraformaldehyde (Solarbio, Wuhan, China) for 15min. Then, the samples were incubated with anti-FUT11 (dilution, 1:100), anti-N-cadherin (dilution, 1:100), anti-E-cadherin (dilution, 1:100), anti-HIF1α (dilution, 1:100) and anti-PDK1 (dilution, 1:100) primary antibodies. After washing with PBS, the samples were incubated with FITC conjugated anti-mouse secondary antibodies (Proteintech, Wuhan, China) and Cy3 conjugated anti-rabbit secondary antibodies (Proteintech, Wuhan, China). Nuclei were stained with DAPI (Boster, Wuhan, China). Finally, confocal microscopy or fluorescent microscope was used to collect the images.

### Immunoprecipitation

Cells were lysed in weak RIPA buffer (Proteintech, Wuhan, China) that included a 1% PMSF (Boster, Wuhan, China). The supernatant was centrifuged and the protein was collected. Then, the anti-FUT11 (dilution, 1:50) antibody and IgG (dilution, 1:50; Beyotime Biotechnology, Hangzhou, China) was added for 6h. The A/G agarose beads (Boster, Wuhan, China) was added for 3h. After washing by PBS three times, isolated immunoprecipitates in beads were collected and analyzed using Western blot.

### Mass Spectrometry

Immunoprecipitation strips were cut into different strips and digested with trypsin. After reductive alkylation, trypsin with mass ratio of 1:50 was added and hydrolyzed at 37°C for 20 h. After desalination, the enzymatic hydrolysate was lyophilized and re-dissolved in 0.1% formic acid solution. Mascot algorithm was used to process MS/MS signals. Parameters included variable modification, oxidation (MET), N-acetylation, and hot glutamine (Gln). Maximum leak, peptide quality tolerance, MS/MS tolerance was 0.5 Da. Proteins were identified on the basis of MS/MS data signals with at least one mascot score exceeding the threshold.

### Chromatin Immunoprecipitation

Chromatin immunoprecipitation (ChIP) assays were performed using a ChIP kit (CST, USA) as per the protocol provided by the manufacturer. Briefly, formaldehyde was used to crosslink cells, and the DNA was sonicated to produce sequences of 200–500 bp in length. Immunoprecipitation was conducted using an anti-HIF1α antibody or IgG control. The precipitated DNA was amplified by qRT-PCR.

### Luciferase Assay

After predicting the binding site of HIF1α (also named hypoxia response element, HRE) in the promoter of FUT11 using online database JASPAR (http://jaspar.binf.ku.dk/), dual luciferase reporter assay was performed to verify the bind. Full‐length FUT11 promoter sequence and corresponding truncated fragment without HRE were carried into the psi‐basic luciferase reporter vector (Promega, USA). Finally, a total of 1×10^4^ PANC-1 and AsPC-1 cells were plated into 24-well plate and cultured overnight at 37°C. Then, both of luciferase reporter vectors contained full‐length FUT11 promoter sequence and corresponding truncated fragment without HRE, and the si‐HIF1α/si‐NC were co-transfected into PC cells using lipidosome 2000 (Solarbio, Wuhan, China). The luciferase activity of cells was determined after transfection at 24h in PC cells cultured in normoxia or hypoxia.

### Statistical Analysis

SPSS software (version 21.0) was employed to perform statistical analysis. The difference between two groups was analyzed using paired t-test, while the difference among multiple groups were determined based on one-way analysis of variance. P*<0.05 was used as a cut-off to consider statistical significance.

## Results

### FUT11 Is a Crucial Hypoxia‐Related Gene and Up-Regulated in PC Tissues

**Figure 1:**
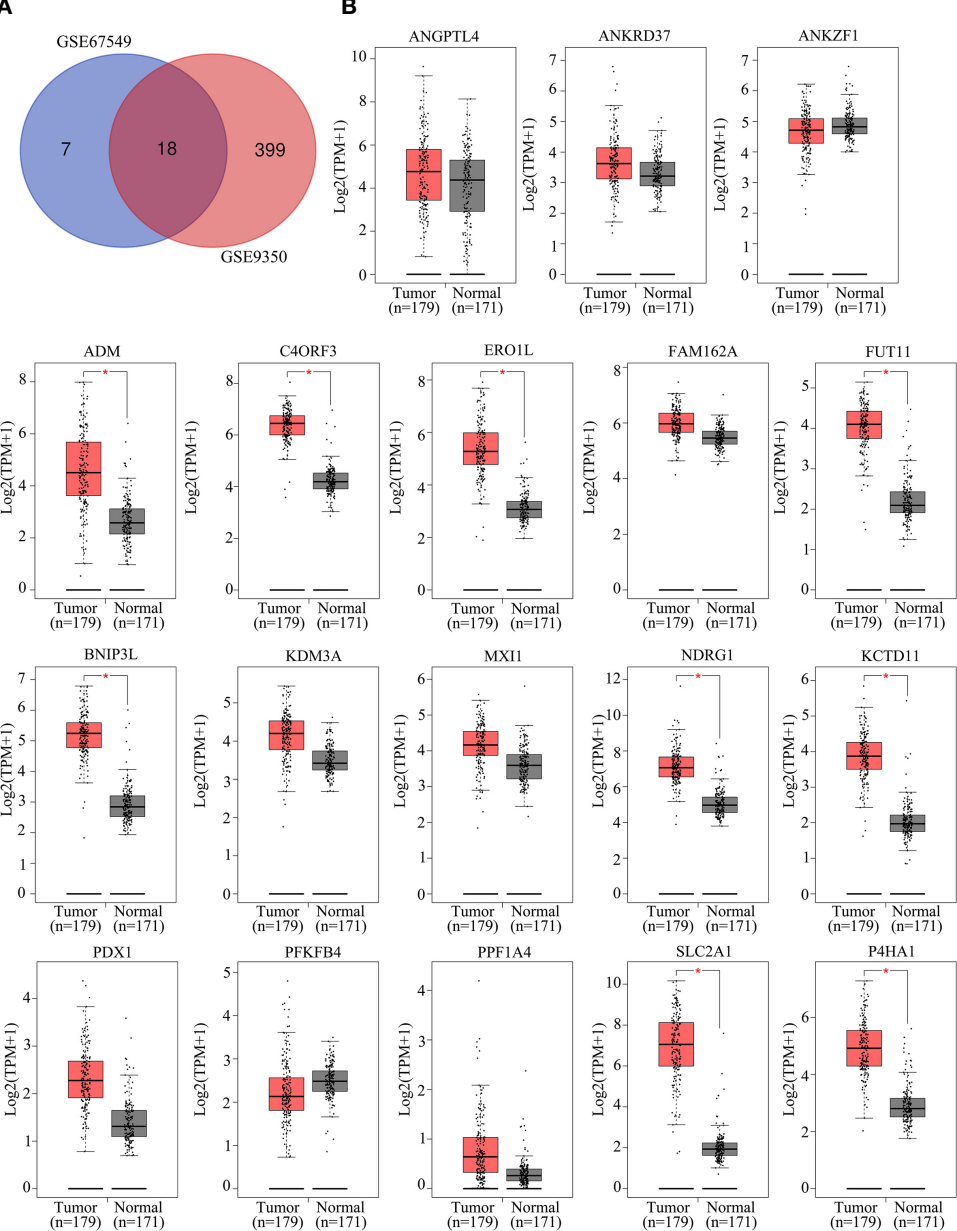
Identification of key hypoxia-related genes in PC. **(A)** Intersection analysis of differentially expressed genes in PC samples under hypoxia based on the gene expression profiles of GSE67549 and GSE9350. **(B)** The mRNA expression of ANGPTL4, ANKRD37, ANKZF1, ADM, C4ORF3, ERO1L, FAM162A, FUT11, BNIP3L, KDM3A, MXI1, NDRG1, KCTD11, PDX1, PFKFB1, PPF1A4, SLC2A1 and P4HA1 in PC tissues compared with non-tumor tissues analyzed by GEPIA online tool.

**Figure 2:**
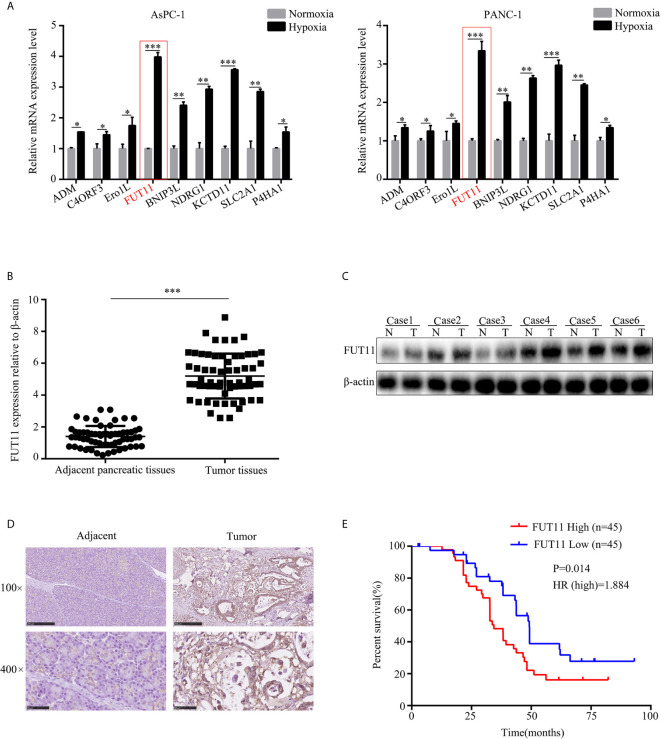
FUT11 was up-regulated in PC and predicted poor outcome. **(A)** qRT-PCR analysis on the mRNA expression of ADM, C4ORF3, ERO1L, FUT11 BNIP3L, KDM3A, MXI1, NDRG1 and KCTD11 in AsPC-1 and PANC-1 cells under normoxia and hypoxia. **(B)** qRT-PCR analysis of mRNA level of FUT11 in 62 pairs of matched PC tissue and adjacent normal tissues. **(C)** Western blot analysis of protein level of FUT11 in PC tissue and adjacent normal tissues. **(D)** IHC analysis of the protein level of FUT11 in pancreatic cancer and normal pancreatic tissue. **(E)** 90 patients with PC were divided into high- and low-expression groups based on the expression of FUT11. Kaplan survival curve showed the overall survival of high FUT11(red) and low FUT11(blue) expression group. *P < 0.05; **P < 0.01; ***P < 0.001.

To identify the key hypoxia-related genes, two gene data profile of PC cells (GSE67549 and GSE9350) under normoxia and hypoxia was analyzed. The results showed that there were 18 common genes differentially expressed in PC cells in hypoxia compared with that in normoxia (**Figure 1A**). Among them, the mRNA levels of ADM, C4orf3, ERO1L, FUT11, BNIP3L, NDRG1, KCTD11, SLC2A1, and P4HA1 were increased in pancreatic cancer tissues compared to adjacent pancreatic tissues from TCGA and GTEx database (**Figure 1B**). These 9 genes were considered as key hypoxia-related genes that involved in the progression of PC. We confirmed this result in the AsPC-1 and PANC-1 cells under normoxia and hypoxia. It was found that all the mRNA levels of these 9 genes were increased under hypoxia compared with normoxia. Among them, a gene named FUT11 increased the most (>3 fold) in AsPC-1 and PANC-1 cells under hypoxia (**Figure 2A**).

We then evaluated the mRNA and protein levels of FUT11 in human PC tissues and adjacent pancreatic tissues. The results showed that the mRNA and protein levels of FUT11 was higher in pancreatic cancer tissues than that in adjacent pancreatic tissues (**Figures 2B–D**). We analyzed the correlation between FUT11 expression and the PC clinical pathology features, and found that FUT11 expression was positively correlated with tumor size (cm), lymph node metastasis, TNM stage, perineural invasion, blood vessel invasion and distant metastasis as shown in **Supplementary Table 2**. We split the patients into high and low FUT11 expression groups based on the expression of FUT11 with the median value of 5.6. The expression of FUT11>5.6 was defined as high expression, while the expression <5.6 was defined as low expression. Kaplan–Meier analysis show that the patients with higher FUT11 expression had a worse prognosis than those with lower FUT11 expression (*P*=0.014, HR=1.884) (**Figure 2E**).

### Suppression of FUT11 Decreased PC Cell Proliferation and Migration in Normoxia and Hypoxia *In Vitro*

**Figure 3:**
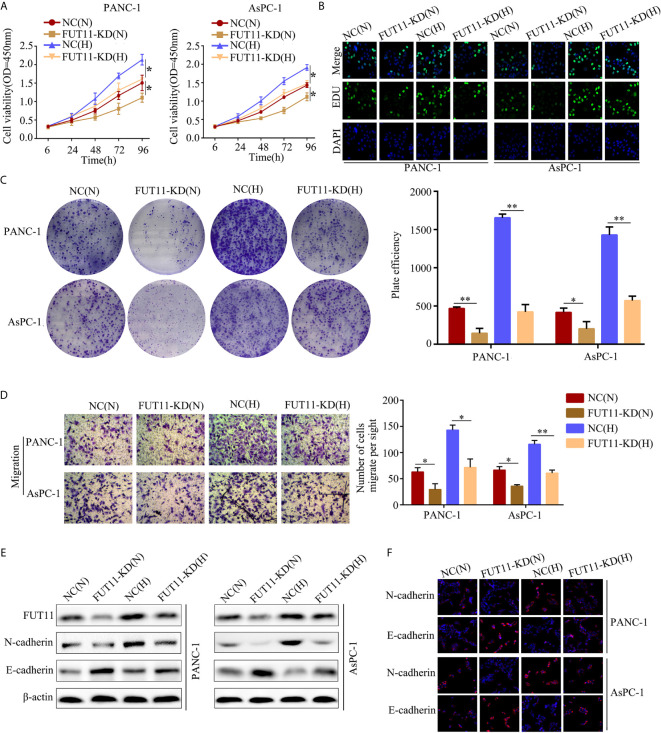
FUT11 regulated the proliferation and migration of PC cells under normoxia and hypoxia *in vitro*. Targeted FUT11 lentivirus and negative control lentivirus were used to construct FUT11 knockdown cells and negative control cells, that were cultured either in normoxia or hypoxia. Cells were divided into four groups: NC(N), cells transfected with negative lentiviral and cultured in normoxia; FUT11-KD(N), cells with FUT11 knockdown and cultured in normoxia; NC(H), cells transfected with negative lentiviral and cultured in hypoxia; FUT11-KD(H), cells with FUT11 knockdown and cultured in hypoxia. **(A)** The effect of FUT11 on PC cell proliferation detected by CCK-8 assay. **(B)** The effect of FUT11 on PC cell proliferation detected by EDU assay. **(C)**The effect of FUT11 on PC cell colony forming ability detected by colony formation assay. **(D)** The effect of FUT11 on PC cell migratory ability detected by Transwell assays. **(E)** Western blotting used to detect the protein level of N-cadherin and E-cadherin of each group. **(F)** Immunofluorescent staining on the expression of N-cadherin and E-cadherin of each group. *P < 0.05; **P < 0.01.

In order to uncover the effects of FUT11 on PC cells in normoxia and hypoxia, targeted FUT11 lentivirus was used to construct FUT11 knockdown cells. CCK-8 assay and EDU assay results showed that FUT11 knockdown reduced the growth of AsPC-1 and PANC-1 cells under normoxia, as well as decreasing the stimulative impact of hypoxia (**Figures 3A, B**). Simultaneously, suppression of FUT11 reduced the colony formation of AsPC-1 and PANC-1 cells in normoxia, as well as reducing the stimulative effects of hypoxia on colony formation (**Figure 3C**). Transwell assay indicated that decreased the expression of FUT11 in AsPC-1 and PANC-1 cells reduced the migratory ability of the cells under normoxia and hypoxia (**Figure 3D**). Western blot assay and immunofluorescence staining demonstrated that FUT11 knockdown decreased N-cadherin protein expression and increased E-cadherin protein expression levels in PC cells under normoxia and hypoxia (**Figures 3E, F**). Taken together, these results suggested that as a key hypoxia-related gene, FUT11 had the potential to regulate the proliferation and migratory ability of PC cells in normoxia and hypoxia condition.

### Knockdown of FUT11 Inhibits the PC Cells Proliferation and Metastasis *In Vivo*

**Figure 4:**
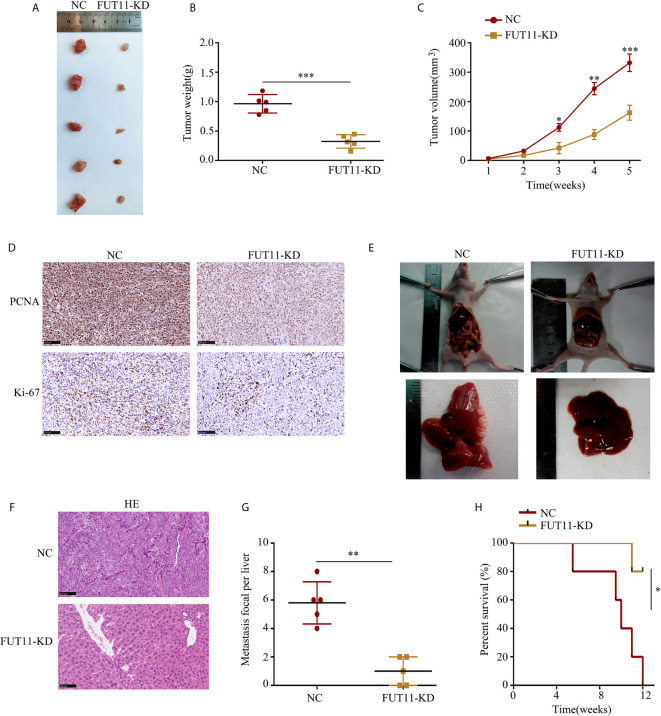
Inhibition of FUT11 suppressed the proliferation and metastasis of PANC-1 cells *in vivo*. **(A)** Representative image of tumor tissues in negative control group and FUT11 knockdown group. **(B)** The mean tumor weight of FUT11 knockdown and negative control groups. **(C)** The proliferative rate of tumor tissues with FUT11 knockdown and negative control. **(D)** IHC staining of Ki-67 and PCNA protein expression in transplanted tumors under different experimental conditions. **(E)** Live metastatic tumor model (n=5). **(F)** IHC staining and HE images showing the metastatic foci in liver in indicated groups. **(G)** Statistical analysis of the average numbers of visible liver metastatic foci. **(H)** Kaplan–Meier survival curves for each experimental group, (n=5). *P < 0.05; **P < 0.01; ***P < 0.001.

The effects of FUT11 knockdown *in vivo* was also determined. We found that tumor tissues derived from FUT11 knockdown cells showed slower growth rate and lower tumor weight than that derived negative control cells (all P<0.05, **Figures 4A–C**). We further assessed the protein expression of proliferation biomarkers KI67 and PCNA in tumor tissues. Results indicated that KI67 and PCNA was decreased in the tumor tissues with low FUT11 expression (**Figure 4D**). The effects of FUT11 on the hepatic metastasis of PANC-1 cells was evaluated in by injecting the FUT11 knockdown and negative control PANC-1 cells into the spleen capsule. Results showed that FUT11 knockdown significantly reduced the metastatic foci in the liver (**Figures 4E–G**). Furthermore, the FUT11 knockdown group had a markedly the longer survival time than the negative control group according to the survival analyses (**Figure 4H**).

### FUT11 Co-Localized With PDK1 in PC Cells and Regulated the Expression of PDK1 *via* Maintaining Its Stability Under Hypoxia

**Figure 5:**
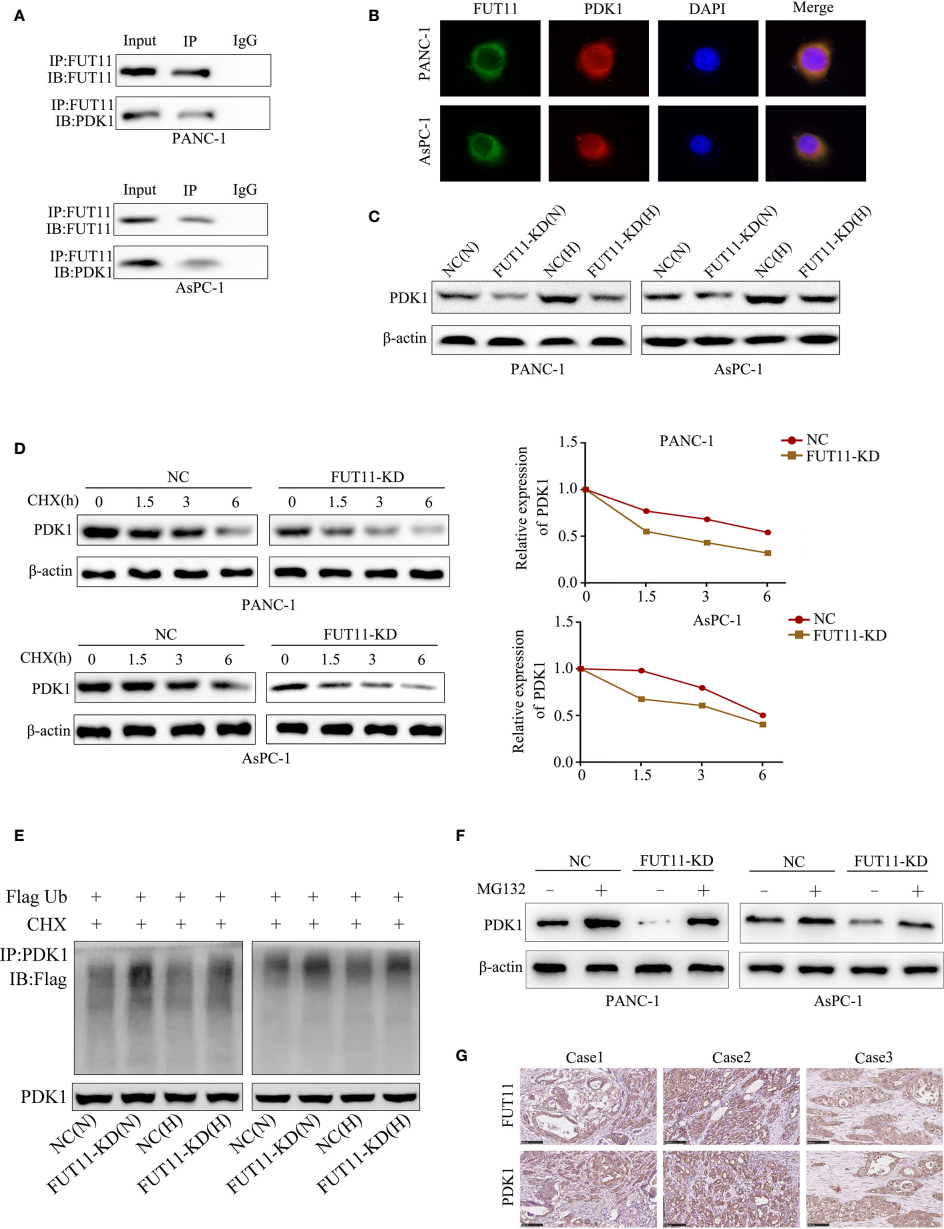
FUT11 bound to PDK1 and regulated its expression. **(A)** Immunoprecipitation on the binding between FUT11 and PDK1. **(B)** Immunofluorescence showed FUT11 co-localized with PDK1 in PC cells.**(C)** Western blot on the expression of FUT11 in sh-scramble and FUT11 knockdown PC cells under normoxia and hypoxia. **(D)** The ubiquitination assay showed the ubiquitination level of PDK1 in sh-scramble and FUT11 knockdown PC cells under normoxia and hypoxia. **(E)** CHX was used to inhibit the protein synthesis, and the degradation of PDK1 in sh-scramble and FUT11 knockdown cells under hypoxia detected using Western blot. **(F)** Western blot on the expression of PDK1 in sh-scramble and FUT11 cells treated with MG132 under hypoxia. **(G)** IHC images showed the co-expression of FUT11 and PDK1.

In order to explore the molecular mechanism of FUT11 in PC development, we used immunoprecipitation with mass spectrometry analysis to determine proteins interacting with FUT11. A total of 700 proteins were found to interact with FUT11 as shown in **Supplementary Table 3**. Among the 700 interacted proteins with FUT11, PDK1 has been revealed as an oncogene in pancreatic cancer, indicating that the effect of FUT11 on PC cells might be associated with PDK1. Immunoprecipitation (IP) and immunofluorescence (IF) analysis demonstrated that FUT11 directly bound to and co-localized with PDK1 (**Figures 5A, B**). The suppression of FUT11 prominently decreased the protein level of FUT11 under normoxia and hypoxia in AsPC-1 and PANC-1 cell (**Figure 5C**). A previous study showed that the members of FUT family can regulate the expression of related proteins by stabilizing their binding proteins and decreasing their ubiquitination (13). Therefore, we considered that FUT11 may bind to PDK1 and protect it from degradation. We used cycloheximide (CHX) to inhibit the synthesis of protein and detect the degradation of PDK1. The results showed that the degradation of PDK1 was increased in FUT11 knockdown cells (**Figure 5D**). To investigate whether FUT11 protect PDK1 *via* inhibiting ubiquitination, we performed the ubiquitination assay. The results showed that the suppression of FUT11 increased PDK1 ubiquitination under normoxia and hypoxia in AsPC-1 and PANC-1 cell (**Figure 5E**). Moreover, it is interesting that treatment with MG132 (10μM) restored the reduction of PDK1 induced by FUT11 suppression under hypoxia (**Figure 5F**). Furthermore, the expression of FUT11 and PDK1 was investigated in our PC clinical samples, results indicated that both FUT11 and PDK1 co-expressed in the PC tissues (**Figure 5G**).

### PDK1 Overexpression Under Hypoxia Decreased the Inhibitory Effect of FUT11 Knockdown

**Figure 6:**
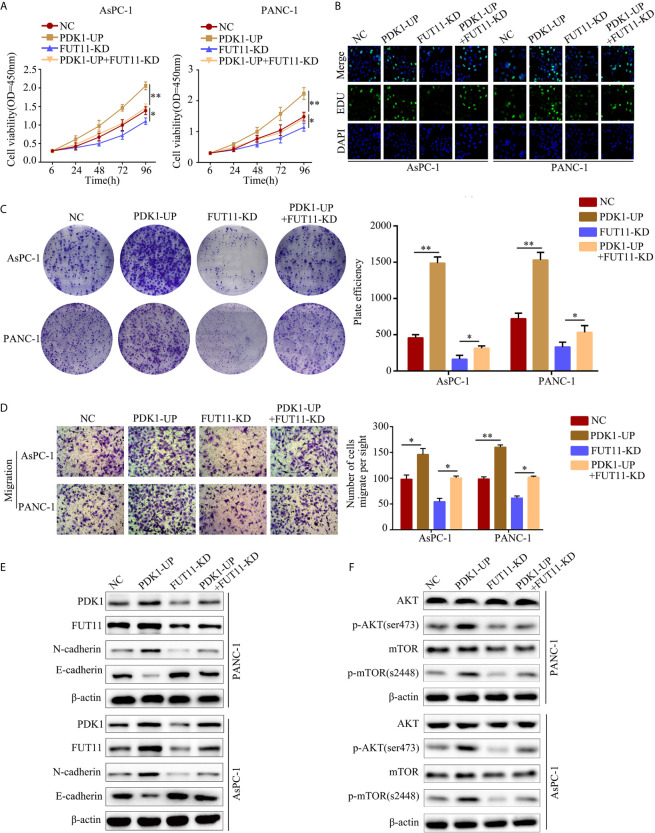
Overexpression of PDK1 reversed the inhibitory effect of FUT11 inhibition on PC cell proliferation and migration. Cells were divided into four groups: negative control group (NC), PDK1 overexpressed group (PDK1-UP), FUT11 knockdown group (FUT11-KD) and PDK1 overexpressed plus FUT11 knockdown group (PDK1-UP + FUT11-KD). All cells were cultured in hypoxia. **(A)** CCK-8 assay on cell viability of each group. **(B)** EDU assay on cell proliferation of each group. **(C)** Colony formation on the colony forming ability of cells in each group. **(D)** Transwell assay on the cell migratory ability of each group. **(E)** Western blot on the protein level of FUT11, PDK1, N-cadherin and E-cadherin in each group. **(F)** Western blot on the protein level of phosphorylated AKT, AKT, phosphorylated mTOR and mTOR in each group. *P < 0.05; **P < 0.01.

To verify whether FUT11 promoted the proliferation of pancreatic cancer *via* increasing the expression of PDK1, we overexpressed PDK1 in FUT11 knockdown PC cells. Then, we used CCK-8 and EDU assays to monitor the cell viability. The results indicated that increased the expression of PDK1 significantly increased the proliferation of FUT11 low-expressed PC cells (**Figures 6A, B**). In addition, the colony formation ability of the cells co-transfected with targeting FUT11 lentivirus and PDK1 overexpression lentivirus under hypoxia was higher than cells transfected with targeting FUT11 lentivirus alone (**Figure 6C**). Similarly, PDK1 overexpressed in FUT11 knockdown PC cells remarkably increased the migratory ability of PC cells (**Figure 6D**). Results of Western blot showed that overexpressed PDK1 in FUT11 knockdown cells significantly increased the protein level of N-cadherin and decreased the protein level E-cadherin (**Figure 6E**). Furthermore, western blot demonstrated the PDK1 significantly activated the AKT/mTOR pathway, while overexpressed PDK1 in FUT11 knockdown cells significantly reversed the inhibitory effects of FUT11 knockdown on the activation of AKT/mTOR pathway (**Figure 6F**).

### FUT11 Was a Target Gene of HIF1α

**Figure 7:**
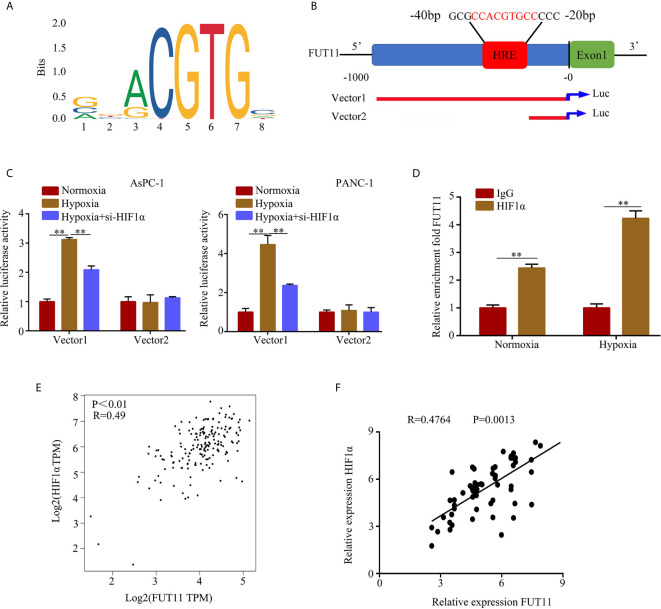
HIF1α regulates the expression of FUT11 by binding to the HRE of the FUT11 promoter. **(A)** The motif of HIF1α. **(B)** The hypoxia response element (HRE) in the promoter of FUT11 (Vector 1: vector with full-length FUT11 promoter; Vector 2: vector with truncated FUT11 promoter which lack of HRE). **(C)** PANC‐1 and AsPC-1 cells transfected with either a full‐length or truncated FUT11 promoter‐pGL3 reporter vector and cultured under hypoxia, with or without si‐HIF1α. After 48 hours, luciferase activity was measured using the dual‐luciferase reporter assay system. **(D)** ChIP assays with anti-HIF1α antibody verifying the binding between HIF1α and hypoxia response element of the FUT11 promoter under normoxia and hypoxia. **(E)** Co-expression of FUT11 and HIF1α based on the data from PC tissues *via* online database GEPIA. **(F)** Co-expression of FUT11 and HIF1α based on the data from our clinical PC tissues (n=62). **P < 0.01.

Hypoxia-inducible factors including HIF1α were the most direct hypoxia response elements. To explore the regulatory network of FUT11, we further determined whether FUT11 was directly regulated by HIF1α. After obtaining the motif of HIF1α in JASPAR database (**Figure 7A**). we found that there is a hypoxia-responsive element (HRE) in the promoter of FUT11 (**Figure 7B**). The results indicated that compared with the control group, hypoxia significantly increased the luciferase activity in the cells transfected with the vector contained full-length FUT11 promoter, while the lack of HRE reduced the luciferase activity. Furthermore, inhibition of HIF1α reversed hypoxia-induced luciferase activity (**Figure 7C**). Anti-HIF1α antibody was enrolled to perform ChIP assays in PANC-1 cells. Results indicated that the HRE in the FUT11 promoter was the major region mediating HIF1α-induced transcription (**Figure 7D**). In addition, it is interesting that FUT11 was co-expressed with HIF1α in TCGA PC samples (**Figure 7E**) and our clinical PC samples (**Figure 7F**).

### Restoration of FUT11 Reversed the Inhibitory Effects of HIF1α Knockdown on PC Cells

**Figure 8:**
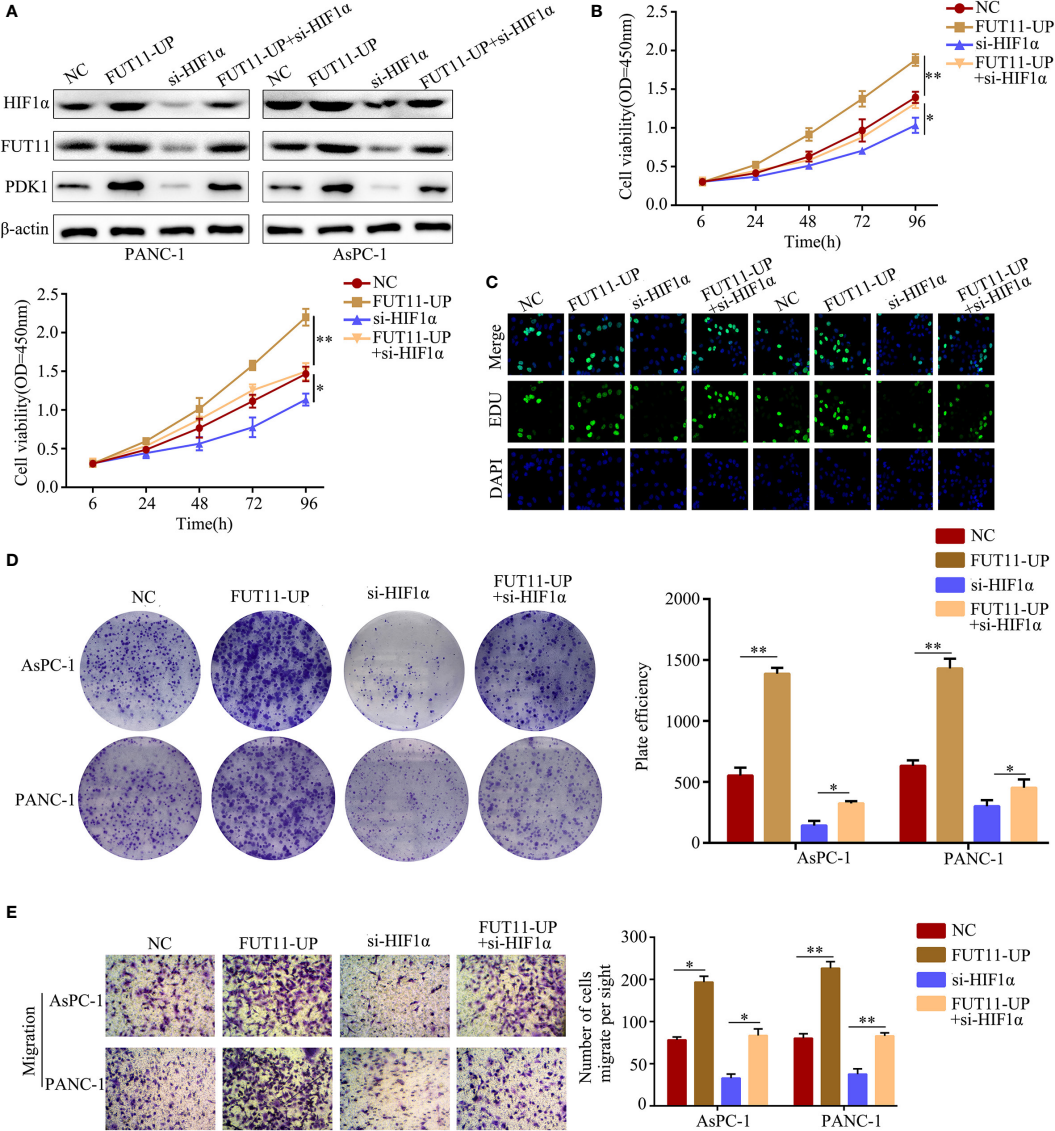
Overexpression of FUT11 reversed the inhibitory effects of HIF1α knockdown on PC cell proliferation and migration under hypoxia. PANC-1 and AsPC-1 cells were divided into four groups: negative control (NC); FUT11 overexpression (FUT11-UP); HIF1α inhibition (si-HIF1α); HIF1α inhibition plus FUT11 overexpression (si-HIF1α+FUT11-UP). All groups of cells were cultured in hypoxia. **(A)** Western blot on the expression of HIF1α and FUT11 in each group of cells. **(B)** CCK-8 assay on the cell viability in each group of cells. **(C)** EDU assay on the proliferation in each group of cells. **(D)** Colony formation assay on the colony forming ability in each group of cells. **(E)** Transwell assays on the migratory ability in each group of cells. The data were shown as means ± S.D. of three independent assays. *P < 0.05, **P < 0.01.

To determine whether FUT11 was involved in the biological function of PC cells induced by HIF1α under hypoxia, we constructed negative control cells, FUT11 overexpressed cells, HIF1α knockdown cells and FUT11 overexpressed plus HIF1α knockdown cells, and cultured them under hypoxia (**Figure 8A**). CCK-8 and EDU assays results showed that suppression of HIF1α inhibited the proliferation of PC cells under hypoxia, while overexpression of FUT11 in HIF1α knockdown cells relieved the suppressive effects of HIF1α knockdown on cell growth (**Figures 8B, C**). Similarly, the colony number of cells with HIF1α inhibition was obviously decreased. Overexpression of FUT11 in HIF1α knockdown cells relieved the inhibitory effects of HIF1α knockdown on colony forming ability under hypoxia (**Figure 8D**). Furthermore, transwell assays demonstrated that HIF1α suppression remarkable decreased the migratory ability of PC cells in hypoxia, while overexpression of FUT11 in HIF1α knockdown cells reversed the inhibitory effects of HIF1α knockdown on cell migration (**Figure 8E**).

## Discussion

Although the therapy for PC had been improved significantly, the prognosis of patients with PC was still poor (15). Moreover, due to early metastasis, most PC patients lost the best time for treatment. Recently, increasing evidences showed that the distant metastasis in early stage of PC cells were driven by signals from tumor environment, including hypoxia (16). Therefore, uncovering the mechanism of hypoxia-regulated response in PC cells is critical for the treatment of PC.

Previous studies had revealed that bioinformatics is a powerful tool to identify genes associated with the development of tumors, including PC (17). Furthermore, online database GEO and TCGA storing thousands of gene database of tumor tissues that provide enough analytical data. In the current study, we used the bioinformatics tool to identify novel hypoxia-related genes. Through analyzing the gene expression profile, we found 18 genes were differentially expressed between hypoxic and normoxic PC samples. Among these 18 genes, 9 of them including ADM, C4orf3, ERO1L, FUT11, BNIP3L, NDRG1, KCTD11, SLC2A1 and P4HA1 were highly expressed in PC tissues. Furthermore, FUT11 was increased the most significant in PC cells under hypoxia condition, up-regulated in PC tissues and predicted poor prognosis of PC patients. These findings suggested that FUT11 may be a novel hypoxia-related gene.

The fucosyltransferase (FUT) family are the key enzymes in cell-surface antigen synthesis during various biological processes such as tumor proliferation, metastasis and drug resistance (18, 19). At present, a total of 13 members consisted FUT1 to FUT11, protein O-fucosyltransferase 1 (POFUT1) and POFUT2 were identified. A number of studies had demonstrated that some members of the FUTs play roles as oncogenes in various types of cancers. FUT8 was up-regulated in non-small cell lung cancer and promoted the process of epithelial–mesenchymal transition (20). POFUT1 increased the activity of Notch1 signaling pathway and promoted the progression of colorectal cancer (21). As shown in the previous studies, inhibition of FUTs including FUT11 significantly decreased the expression and activity of ERK1/2 and p38 MAPK pathways, as well as the progression of human invasive ductal carcinoma (22). FUT11 was highly expressed in gynecological cancers, and overexpression of FUT11 in patients predicted poor outcome (23). However, the effect of FUT11 on proliferation and metastasis of human PC cells have not yet been defined. Using CCK-8 assay, colony formation assay and transwell assay, we found that FUT11 inhibition significantly decreased proliferation and migration of PC cells in both hypoxic and normoxic environment. These results were in consistent with previous studies. Via performing xenograft tumor model and *in vivo* metastatic tumor model, we found that FUT11 inhibition decreased the PC cells proliferation and metastasis *in vivo*. These results suggested that FUT11 was linked to hypoxia, because it had the potential to regulate PC cells proliferation and migration under normoxia and hypoxia.

PDK1 has emerged as an important oncogene in many types of cancers including PC (24). Lucero-Acuna A et al. has been reported that the expression of PDK1 is up-regulated in human PC and promotes cancer cell growth and mobility (25). Xia S et al. showed that knockdown of PDK1 forces cells containing activated p21(Ras) to undergo apoptosis in PC cells (26). Previous studies have shown that, one of the targets of PDK-1 was AKT, which can be activated by phosphorylation on two residues (T308 and S473) for full oncogenic activity (27). However, the mechanisms of PDK1 in regulating tumor progression is not clear. In the current study, through immunoprecipitation with mass spectrometry analysis, PDK1 was identified as one of the potential downstream genes of FUT11, which co-expressed with FUT11. Furthermore, using immunoprecipitation and Western blot, we found that FUT11 directly bound to PDK1 and regulated its expression in normoxia and hypoxia. Based on previous studies, FUTs can bind to a series of proteins and maintain their stability *via* blocking the binding site of protease (13). Therefore, we determined whether FUT11 regulated PDK1 *via* maintaining its stability. Consistent with our speculation, knockdown of FUT11 under hypoxia increased the degradation of PDK1. Furthermore, overexpression of PDK1 in PC under hypoxia relieved the inhibitory impacts of FUT11 knockdown on cell proliferation and migration.

The relationship among hypoxia-inducible factors, hypoxia microenvironment, hypoxia related genes and the development of PC were widely reported in previous studies. For example, PAFAH1B2 regulated by HIF1α under hypoxia promoted the growth and mobility of PC cells (28). MTA1 was regulated by HIF-α/VEGF axis and promoted the development of PC (29). Similarly, overexpression of hydroxyproline *via* EGLN/HIF1A is associated with distant metastasis in PC (30). Similarly, our previous study also demonstrated that YEATS2 directly targets HIF1α which promotes PC cell proliferation and mobility (31). In the present study, we provided the first evidence that FUT11 was a novel target gene of HIF1α, which involved in the biological function mediating by HIF1α under hypoxia.

In conclusion, our present study demonstrated that FUT11 is a new hypoxia related gene, and is overexpressed in pancreatic cancer tissues and related to poor prognosis of pancreatic cancer patients. FUT11 is regulated by HIF1α and promotes PC cells proliferation and migration *via* maintaining the stability of PDK1 mediates AKT/mTOR signaling pathway. FUT11 could be an effective target for overcoming the hypoxia response of PC.

## Data Availability Statement

The original contributions presented in the study are included in the article/**Supplementary Material**. Further inquiries can be directed to the corresponding authors.

## Ethics Statement

The studies involving human participants were reviewed and approved by The Ethics Committee of GuiZhou Medical University Ethics. The patients/participants provided their written informed consent to participate in this study. The animal study was reviewed and approved by Animal Experimental Ethical Inspection Form of Guizhou Medical University.

## Author Contributions

WC, ZZ, ZY, and SL contributed to the experiment design, and data analysis. RP, HW, WC, and SL contributed to the experiment implementation, YN, XZ, HC, and SL contributed to manuscript draft and data analysis. All authors contributed to the article and approved the submitted version.

## Conflict of Interest

The authors declare that the research was conducted in the absence of any commercial or financial relationships that could be construed as a potential conflict of interest.

## Supplementary Material

Supplementary Table 1 The primers used for quantitative real-time PCR (qRT-PCR) and plasmid sequence.

Supplementary Table 2 The Association between FUT11 expression and clinicopathological features of PC patients.

Supplementary Table 3 Mass spectrometry of immunoprecipitation proteins interacting with FUT11.
